# Management of perioperative acute massive pulmonary embolism: A case series

**DOI:** 10.1002/ccr3.4078

**Published:** 2021-03-28

**Authors:** Ji‐Yoon Kim, Young‐Seok Lee, Hyun Oh Park, Il Woo Shin

**Affiliations:** ^1^ Department of Anesthesiology and Pain Medicine School of Medicine Kyungpook National University Kyungpook National University Medical Center Daegu South Korea; ^2^ Department of Neurosurgery School of Medicine Kyungpook National University Kyungpook National University Medical Center Daegu South Korea; ^3^ Department of Thoracic and Cardiovascular Surgery Gyeongsang National University Hospital Jinju Korea; ^4^ Department of Anesthesiology and Pain Medicine Gyeongsang National University College of Medicine Jinju Korea; ^5^ Institute of Health Sciences Gyeongsang National University Jinju Korea

**Keywords:** acute pulmonary embolism, echocardiography, extracorporeal membrane oxygenation

## Abstract

The management of acute massive pulmonary embolism during the perioperative period is challenging. Accurate diagnosis using echocardiography and application of rapid extracorporeal membrane oxygenation can improve patients' outcomes.

## Graphical abstract

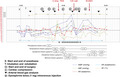

## 1 INTRODUCTION

Acute massive pulmonary embolism is a very serious life‐threatening disease. We present two cases of intraoperative acute massive pulmonary embolism. The patients had cardiac arrest lasting for >30 minutes, but they were resuscitated. Accurate diagnosis using echocardiography and application of rapid extracorporeal membrane oxygenation improved the patients' outcomes.

Acute massive pulmonary embolism (PE) is a very serious life‐threatening condition. Approximately 60% of patients with massive PE experience hypotension, cardiogenic shock requiring catecholamines, and even cardiopulmonary resuscitation (CPR) due to hemodynamic collapse.^1^ The mortality rates in hospitals vary, ranging from 8.1% in patients with a stable condition to 25% in patients with cardiogenic shock and even to 65% in patients necessitating CPR.^1^ Patients in a state of PE are in serious conditions and may die within an hour; thus, rapid diagnosis and treatment is very important.^2^ However, the management of PE is challenging. Its diagnosis is not easy, but echocardiography is in the spotlight as a quick diagnostic method in the perioperative setting. There are various treatment modalities, including anticoagulants, thrombolytic therapies, and surgical embolectomy, reported for PE.^3^, ^4^ However, its treatment is very difficult, because the patients are experiencing circulatory collapse. Extracorporeal membrane oxygenation (ECMO) has great value as a bridging therapy.^3^, ^5^ We described two cases of PE that were successfully managed with transesophageal echocardiography (TEE) and ECMO.

## 2 CASE REPORT

### 2.1 Case 1

**FIGURE 1:**
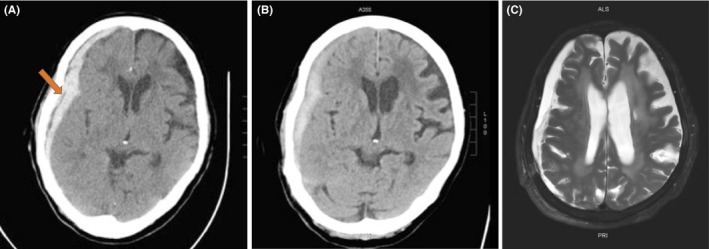
A, Traumatic subdural hematoma on the right temporal area of the patient was confirmed using brain computed tomography (CT) (arrow). B, Brain CT on the 11th day of admission, which was performed just before surgery. There was no significant difference in the hematoma volume observed immediately after admission. C, Brain magnetic resonance imaging (MRI) performed before discharge. Hypoxia‐induced encephalopathy was found along with hydrocephalus

An 86‐year‐old woman (weight, 58 kg; body mass index [BMI], 24.1 kg/m^2^) lost her consciousness for a short period of time when she fell down from a hill while working in the field. She was then brought to the emergency department of our institution. Traumatic subdural hematoma on her right temporal area was confirmed using brain computed tomography (CT) (Figure 1A). Careful history‐taking revealed a history of hypertension managed with medications, diabetes mellitus without medications, and mild dementia. Given that the patient was alert and the size of the hematoma was not large enough to require emergent surgery, she was put on bed rest and received mannitol infusion for 2 days to reduce intracranial pressure.

**FIGURE 2:**
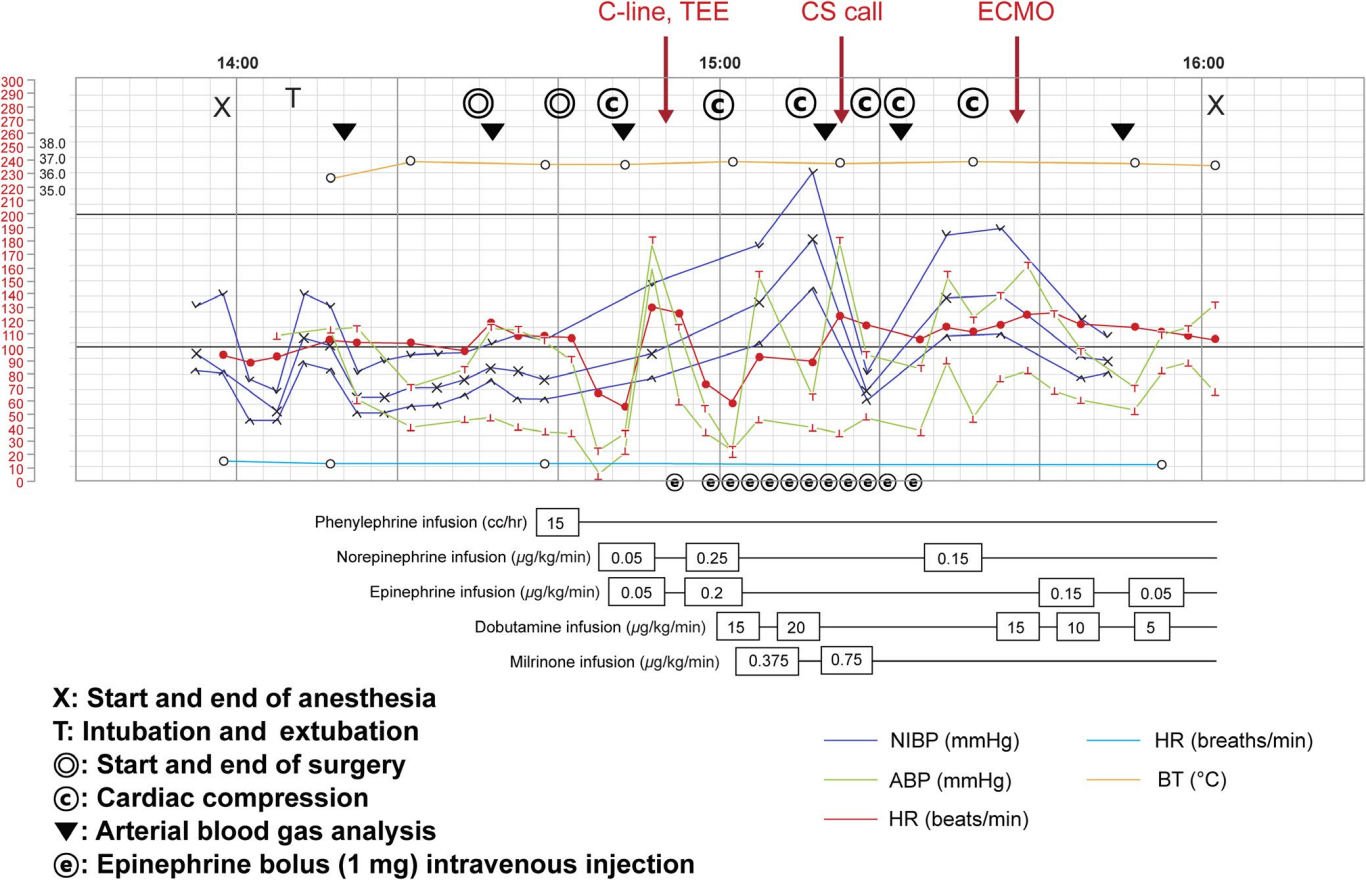
Anesthesia timeline record of the patient showing unstable perioperative vital signs. At 14:40, cardiac compression was performed. At 14:50, central line catheterization and TEE were performed. At 15:15, the cardiothoracic surgeon was called. ECMO insertion was completed at 15:40. ABP, arterial blood pressure (mm Hg); BT, body temperature (°C); HR, heart rate (beats/min); NIBP, noninvasive blood pressure (mm Hg); RR, respiratory rate (breaths/min)

**FIGURE 3:**
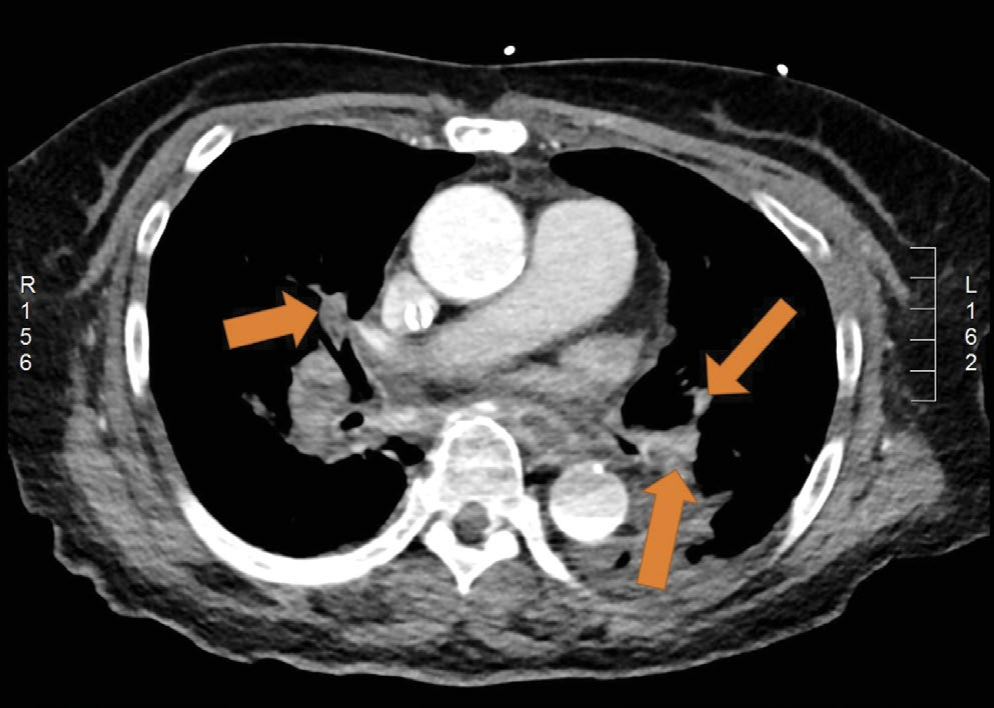
Chest computed tomography (CT) showing multiple pulmonary emboli (arrow)

**FIGURE 4:**
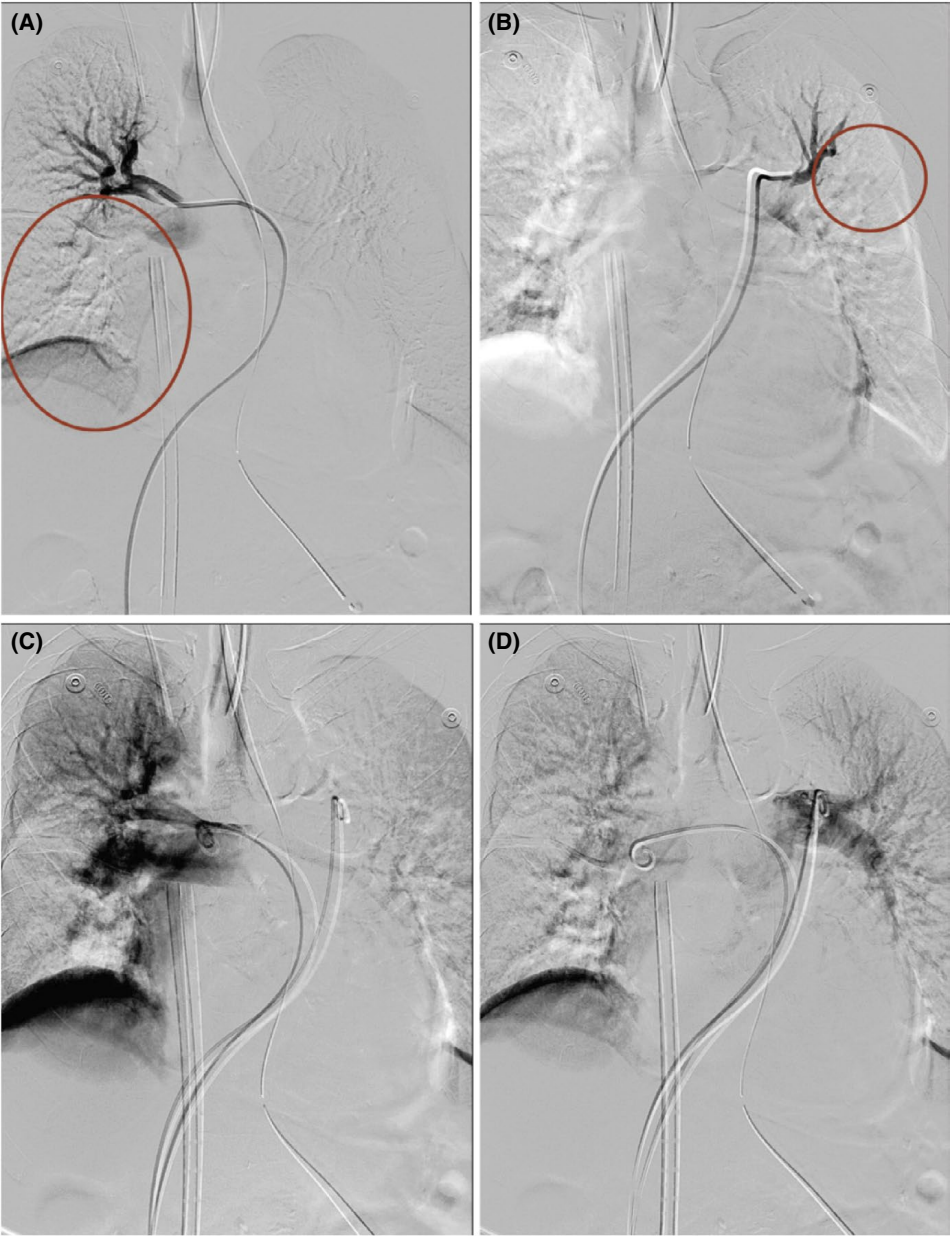
A, Angiography shows pulmonary blood flow reduction (circle) due to the presence of multiple pulmonary emboli. Perfusion only occurs in the right and left upper lobes. B, Angiographic images after catheter‐directed thrombolysis. C and D, The pulmonary blood flow is better than that before the procedure

On the 11th day after her hospitalization, her level of consciousness suddenly declined; thus, an emergency CT was performed. CT revealed no significant increase in the size of the hematoma (Figure 1B). However, because the hematoma still remained, the neurosurgeon decided to perform an emergent burr‐hole craniostomy. At the end of the surgery, the end‐tidal carbon dioxide tension (ETCO_2_) suddenly decreased (from 33 to 14 mm Hg) and hemodynamic collapse occurred (Figure 2). After chest compressions and injection of epinephrine, return of spontaneous circulation (ROSC) was achieved. TEE performed by the anesthesiologists revealed right ventricular dilatation and severe tricuspid regurgitation. Normal lung sounds were heard during auscultation. However, the patient's condition worsened again. CPR was repeated, and ROSC was achieved again. During this time, the arterial blood gas analysis showed hypoxemia and hypercarbia despite the low ETCO_2_ (from 13 to 21 mm Hg) (Table 1). ECMO was considered due to the repeated CPR. Finally, at 40 minutes after the first arrest, ECMO was performed. After the application of ECMO, the patient's vital sign stabilized and she underwent CT angiography, which confirmed the presence of multiple PE (Figure 3). The patient received catheter‐directed thrombolysis with urokinase (Figure 4). She was weaned off from ECMO 2 days later. Unfortunately, the patient developed hypoxia‐induced encephalopathy (Figure 1C) and did not fully recover. She had an altered mental status but could still obey simple commands. The patient was transferred to a secondary hospital for conservative treatment at 89 days after admission.

**TABLE 1:** Arterial blood gas analysis of the patient

Time (FiO_2_/ETCO_2_)	Induction (0.5/26)	Operation start (1.0/36)	1st ROSC (1.0/19)	2nd ROSC (1.0/13)	3rd ROSC (1.0/21)	4th ROSC (1.0/28)	After ECMO
pH	7.37	7.26	7.12	7.04	7.04	6.98	7.28
pCO_2_ (mm Hg)	44	59	66	84	114	97	45
pO_2_ (mm Hg)	60	73	78	49	59	59	157
${\text{HCO}}_{3}^{‐}$ (mmol/L)	25.4	26.5	21.5	22.7	30.8	22.8	21.1
BE (mmol/L)	−0.0	−1.3	−8.3	−8.4	−1.2	−8.7	−5.3
SaO_2_ (%)	90	92	90	63	76	72	99
Glucose (mg/dL)	129	151	312	296	342	330	283
Lactate (mmol/L)	0.8	1.1	4.9	6.4	7.9	7.8	6.7

Abbreviations: ECMO, extracorporeal membrane oxygenation; ETCO_2_, end‐tidal carbon dioxide; FiO_2_, fraction of inspired oxygen; ROSC, return of spontaneous circulation.

### 2.2 Case 2

**FIGURE 5:**
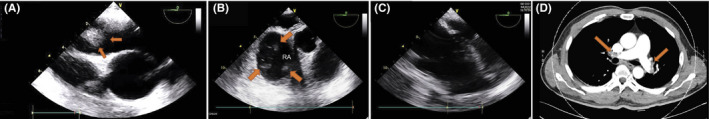
A, In the mid‐esophageal ascending aortic short‐axis view of transesophageal echocardiography, the embolus is clearly visible in the right main pulmonary artery (arrow). B, Mid‐esophageal aortic valve short‐axis view shows multiple emboli in the right atrium. (arrow). C, Mid‐esophageal 4‐chamber view shows dilated right ventricle and right atrium chambers and shifted interventricular septum. D, Chest computed tomography. Large pulmonary emboli (arrow) are seen in both main pulmonary arteries

A 70‐year‐old man (weight, 93 kg; BMI, 31.7 kg/m^2^) was scheduled to undergo open reduction and internal fixation for an ankle fracture under local anesthesia in the operating room. He had a medical history of hypertension and diabetes mellitus. Fifteen days before surgery, the patient had multiple fractures in his lower extremities owing to a car accident and had already undergone two previous surgeries under general anesthesia. While the orthopedic surgeon was preparing for the surgery of the fractured ankle, the patient complained of chest tightness. The patient's systolic blood pressure decreased to 60 mm Hg. The surgeon called an anesthesiologist. The patient had severe hypotension and bradycardia and lost consciousness. The surgeon started performing chest compressions. The anesthesiologists performed tracheal intubation, central vein insertion, arterial insertion, and TEE during chest compressions. TEE showed a prominent thrombus located in the right main pulmonary artery (Figure 5A) and right atrium (Figure 5B). The right side of the heart was also enlarged (Figure 5C). Despite continued chest compressions and inotrope treatment for 45 minutes, the patient still continued to be in a state of shock. We determined that ECMO would be beneficial for the patient. Immediately after the application of ECMO, the patient's vital signs stabilized, and he was transferred to the radiology suite. Chest CT showed large prominent thrombi in both the main pulmonary arteries (Figure 5D). Urokinase was administered via a catheter (Table 2). After 3 days, the patient was weaned off from ECMO, and after 4 days, he was weaned off from the ventilator. On the 35th day of hospitalization, surgery for the ankle fracture was performed under local anesthesia, and the patient was discharged without any complications on the 50th day of hospitalization.

**TABLE 2:** Event timetable of the 2nd case. Due to the unstable vital signs, chest compression was continuously performed. His vital signs were stabilized after extracorporeal membrane oxygenation (ECMO) insertion

Time	Events
16:25	Patient entered the operating room. Initial vital signs (V/S): 120/70 mm Hg‐80 beats per minute‐18 breath per minute, SpO2 98%
16:35	Orthopedic surgeon began preparing for the operation.
17:00	Patient suddenly complained of chest discomfort. V/S checked again: 60/40 mm Hg‐80 beats per minute‐20 breath per minute. Surgeon stopped preparing for surgery and called an anesthesiologist.
17:05	Anesthesiologists arrived. The patient's level of consciousness has deteriorated.
17:10	Intubation. Epinephrine was intravenously administered.
17:15	Cardiopulmonary resuscitation was performed. Arterial and central catheterization and TEE were performed. Prominent thrombi were seen in the main pulmonary artery and right atrium. Cardiothoracic surgeon was called for ECMO insertion.
18:00	ECMO insertion. Vital signs: 140/80 mm Hg‐70 beats per minute‐10 breath per minute.
18:20	Patient was transferred to the radiologic suite for thrombolysis.
19:45	Patient entered the intensive care unit after undergoing a thrombolysis.

## 3 DISCUSSION

Venous thromboembolism (VTE) refers to both thrombosis and embolism that occur in veins. Deep vein thrombosis (DVT) and PE are important clinical presentations of VTE. In most cases, PE results from DVT occurring in the lower extremities (70.8%).^6^ Acute massive PE is the most serious form of VTE.

In 1856, Virchow wrote the principle of thrombus formation. Thrombosis is caused by a vascular injury, blood flow stagnation, and hypercoagulability. This concept has not yet changed. Based on the Virchow's triad, the well‐known risk factors for thrombosis include trauma, spinal cord injury, hospitalization, infection, paralytic stroke, and pregnancy. As in our cases, old age is also a risk factor.^7^ Surgical patients also have many risk factors for PE formation, including inflammation caused by tissue trauma, activation of the clotting cascade, and venous stasis due to immobilization.^4^ The incidence of PE has been reported to be 66‐104 people per 100 000 in the United States and Europe.^8^ However, in the perioperative setting, the incidence of PE increases from 0.3% to 1.6%.^4^

Fortunately, the incidence of PE was estimated to be 15%‐20% lower as compared to Western populations.^9^ However, the recent trend in PE occurrence shows a sharp increase in the incidence in the Asian population because of the rapid increase in the elderly population and the introduction of Western lifestyle.^9^ This phenomenon calls for clinicians' awareness.^9^ Given that preventive measures can play a big role, clinicians should dedicate themselves to the prevention of PE. Early ambulation, application of elastic stocking, and intermittent pneumatic compression are easy methods for preventing PE. Moreover, pharmacological prophylaxis is provided for patients with an increased risk of developing PE. However, it can cause hemorrhage in many cases; thus, its careful application is needed. It is necessary to identify the embolism risk in all patients and to take appropriate action. However, taking preventive actions is not sufficient, especially considering that the prophylaxis failure rate is estimated to be up to 50%.^10^

Acute PE interferes with both circulation and gas exchange. The obstruction of the pulmonary artery causes pressure overload in the right ventricle (RV), leading to RV expansion and inflammatory reactions. The oxygen demand of the RV also increases. As RV ischemia is induced, RV contractility decreases and its output decreases. Consequently, the left ventricle (LV) preload decreases. As the output of LV decreases, hypotension occurs and coronary perfusion decreases. Oxygen supply to the RV becomes limited. This creates a vicious cycle.^7^

Depending on the location of the clots, the clinical symptoms vary from asymptomatic to sudden death. PE is suspected in patients with dyspnea (50.1%), chest pain (39.4%), hemoptysis (22.9%), syncope (5.5%), altered mental status (4.8%), and signs of DVT such as leg swelling (23.5%).^11^ However, these are nonspecific symptoms, and even after general anesthesia, most of these symptoms are masked. As seen in the first case, a sudden decrease in ETCO_2_ is an early sign of a potential PE for patients undergoing general anesthesia and mechanical ventilation.^12^ In both of our cases, the patients complained of intermittent chest pain when they were in the ward. However, the clinicians overlooked the symptoms, and eventually, we experienced these events.

In both cases, TEE played a critical role in diagnosis. Over the past decades, the importance of TEE in perioperative management has increased,^3^ even in the management of PE. CT is known as the “gold standard” for diagnosing PE, but it is practically impossible to transfer a patient with an unstable condition to a CT room. It is not easy to detect an embolism using echocardiography in patients with PE. One study detected an embolism in only 46% of their cases.^13^ However, secondary signs of pulmonary artery obstruction, including leftward bowing of the interatrial septum (98%), RV dysfunction (96%), and at least moderate tricuspid regurgitation (50%), are found in most patients. The McConnell sign (depressed contractility of the RV free wall compared to the relative sparing function of the RV apex) is suggestive of PE.^3^, ^7^, ^13^ Therefore, if a patient undergoing echocardiography is suspected as having PE, clinicians should focus on finding a secondary sign rather than on directly visualizing the PE. The quantitative analysis of TEE is more difficult than transthoracic echocardiography. Tricuspid annular plane systolic excursion (TAPSE) is one of the verified methods for assessing RV systolic function. In TEE, the modified TAPSE can be considered an alternative method for evaluating the RV systolic function, especially at the busy operating room.^14^

If an accurate diagnosis has been made, veno‐arterial ECMO may be helpful for patients with hemodynamic collapse. It may be possible to prevent end‐organ damage, thereby improving patients' survival.^7^ The overall mortality in patients with massive PE who received ECMO support was 5%‐40%.^15^, ^16^, ^17^ The causes of mortality were catheter malposition (eg, arterial to arterial) and end‐organ damage that had already occurred before ECMO application.^5^ Long‐term application of ECMO as treatment for PE alone is not desirable. ECMO can lead to serious complications, such as infection, bleeding, and leg ischemia.^18^ ECMO should be used very selectively and in a short period of time.^3^, ^5^ Studies reporting good survival suggest that the ECMO application period should be 5.1 days.^17^ Decannulation should be performed when the burden from the thrombus is reduced.^17^ However, in patients with continued hemodynamic instability after ECMO, despite the use of high doses of vasopressors, surgical embolectomy may be considered as the last resort.^5^

In conclusion, accurate diagnosis and quick response are important for managing intraoperative acute massive PE. In all cases, ECMO is not the answer, but if hemodynamic collapse persists despite adequate treatment, prompt application of ECMO can result in better patient prognosis.

## CONFLICT OF INTEREST

None declared.

## AUTHOR CONTRIBUTION

JYK: drafted and wrote the manuscript. YSL and HOP: performed the operation and contributed to patient care. IWS: revised and approved the manuscript.

## ETHICAL APPROVAL

This study was approved by the Institutional Review Board of the Gyeongsang National University Hospital (IRB No: 2020‐01‐031).

## Data Availability

In accordance with the “DFG Guidelines on the Handling of Research Data,” we will make all data (digitized electrophysiological recordings [IGOR wave metrics format]); confocal images (a.o. TIFF format) available upon request. The data set will be archived for at least 10 years after publication.
